# High-resolution diffusion tensor imaging of the human kidneys using a free-breathing, multi-slice, targeted field of view approach

**DOI:** 10.1002/nbm.3190

**Published:** 2014-09-15

**Authors:** Rachel W Chan, Constantin Von Deuster, Christian T Stoeck, Jack Harmer, Shonit Punwani, Navin Ramachandran, Sebastian Kozerke, David Atkinson

**Affiliations:** a Centre for Medical Imaging, University College LondonLondon, UK; b Institute for Biomedical Engineering, University and ETH ZurichZurich, Switzerland; c Division of Imaging Sciences, King's College LondonLondon, UK; d Radiology Department, University College London HospitalsLondon, UK

**Keywords:** human kidney, diffusion tensor imaging, targeted field of view, free breathing, multi-slice imaging

## Abstract

Fractional anisotropy (FA) obtained by diffusion tensor imaging (DTI) can be used to image the kidneys without any contrast media. FA of the medulla has been shown to correlate with kidney function. It is expected that higher spatial resolution would improve the depiction of small structures within the kidney. However, the achievement of high spatial resolution in renal DTI remains challenging as a result of respiratory motion and susceptibility to diffusion imaging artefacts. In this study, a targeted field of view (TFOV) method was used to obtain high-resolution FA maps and colour-coded diffusion tensor orientations, together with measures of the medullary and cortical FA, in 12 healthy subjects. Subjects were scanned with two implementations (dual and single kidney) of a TFOV DTI method. DTI scans were performed during free breathing with a navigator-triggered sequence. Results showed high consistency in the greyscale FA, colour-coded FA and diffusion tensors across subjects and between dual- and single-kidney scans, which have in-plane voxel sizes of 2 × 2 mm^2^ and 1.2 × 1.2 mm^2^, respectively. The ability to acquire multiple contiguous slices allowed the medulla and cortical FA to be quantified over the entire kidney volume. The mean medulla and cortical FA values were 0.38 ± 0.017 and 0.21 ± 0.019, respectively, for the dual-kidney scan, and 0.35 ± 0.032 and 0.20 ± 0.014, respectively, for the single-kidney scan. The mean FA between the medulla and cortex was significantly different (*p* < 0.001) for both dual- and single-kidney implementations. High-spatial-resolution DTI shows promise for improving the characterization and non-invasive assessment of kidney function. © 2014 The Authors. *NMR in Biomedicine* published by John Wiley & Sons, Ltd.

## INTRODUCTION

Diffusion-weighted imaging (DWI) is a non-invasive MRI technique that is sensitive to the Brownian motion of water molecules and restrictions of this motion. It has broad clinical applications and is becoming increasingly important in cancer imaging, where it is useful for the characterization of lesions. DWI does not require any intravenous contrast agent administration and thus is suitable for the imaging of patients with kidney dysfunction, for which the use of contrast can be contraindicated. Although DWI primarily looks at the presence of restricted diffusion, diffusion tensor imaging (DTI), with more measurements, can also probe the principal directions of diffusion and its anisotropic nature.

Anisotropic diffusion in the human kidney has been demonstrated with DTI by Ries *et al*. 1. This and other studies 2–6 have revealed differences in the fractional anisotropy (FA) between the medulla and cortex of healthy kidneys, as well as radial diffusion patterns in the healthy medulla. In kidney disease, several studies have shown that DTI can non-invasively reveal information regarding kidney function, for example, in chronic parenchymal diseases 7, can detect early changes in diabetic nephropathy 8 and, in particular, can provide correlates on the function of renal allografts after kidney transplant 9,10. These studies have found that renal impairment can lead to lower medullary FA values relative to healthy kidneys.

The achievement of high spatial resolution in renal DTI remains challenging. Image resolution has been limited by respiratory motion, the large fields of view used in the abdomen and the long readouts in diffusion imaging which are susceptible to off-resonance and eddy current artefacts. However, higher spatial resolution is expected to improve the characterization and depiction of small structures within the kidney, including the medullary pyramids and the thin cortical layer. One method to achieve higher spatial resolution is to employ a reduced field of view (RFOV) or targeted field of view (TFOV), such as in Jin *et al*. 11 for kidney DWI. However, this method is limited to single-slice acquisitions and has not been used in DTI. A multi-slice RFOV approach 12 has been demonstrated in the spine, but is less suitable for the imaging of moving organs, such as the kidneys, because two refocusing pulses are required for each slice.

The purpose of this study was to obtain, in healthy subjects, high-resolution FA maps and colour-coded diffusion orientations of the human kidney over multiple contiguous slices using a free-breathing protocol. A multi-slice TFOV DTI technique 13,14 was used and two implementations are presented: a dual-kidney TFOV ‘D’ and a single-kidney TFOV ‘S’. The mean medullary and cortical FAs were qualitatively and quantitatively assessed over the entire kidney volume.

## METHODS

### MRI

Written informed consent was obtained from 12 healthy subjects (eight men, four women; mean age, 28 years; age range, 24–36 years), who were imaged under a protocol approved by the local institutional review board. Scans were performed on a 3-T Philips Achieva TX system (Philips Healthcare, Best, the Netherlands). Subjects were scanned with a TFOV spin-echo diffusion sequence that used a non-coplanar application of excitation and refocusing pulses, combined with outer volume suppression [previously demonstrated in the spinal cord 13,14 and prostate 15] to achieve RFOVs with multiple contiguous slices. Two implementations, the dual-kidney (TFOV D) and single-kidney (TFOV S) TFOV sequences, were used to acquire angulated coronal slices throughout the kidneys. TFOV D simultaneously imaged both kidneys, whereas TFOV S was optimized for targeted imaging over a single kidney, which allowed a higher in-plane resolution with a voxel size of 1.2 × 1.2 mm^2^ (compared with 2 × 2 mm^2^ when imaging both kidneys). The body mass index (BMI = *W*/*H*^2^) of the subjects ranged from 18.3 to 28.1 kg/m^2^, with an average of 23.2 ± 3.1 kg/m^2^.

Each subject was scanned with TFOV D (TR/TE = 5000/80 ms; FOV, 220 × 126 mm^2^; superior–inferior (SI) phase-encoding (PE) direction; 12 slices; slice thickness, 5.5 mm; two signal averages; matrix, 112 × 112; readout duration, 81 ms), followed by a scan with TFOV S in which a single kidney, either the right or left, was imaged (TR/TE = 5000/106 ms; FOV = 120 × 72 mm^2^; left–right (LR) PE direction; eight slices; slice thickness, 5.5 mm; three signal averages; matrix, 112 × 112; readout duration, 106 ms). The FOV was increased by 5–20 mm to include larger kidney sizes in some subjects in both TFOV D and S. In TFOV S, the right kidney was imaged in six subjects and the left kidney in the six other subjects. Each scan consisted of 15 diffusion-encoding directions with *b* = 450 s/mm^2^ and a *b* = 0 s/mm^2^ image. The TFOV technique consisted of integrated saturation slabs in the PE direction (on both sides of the FOV) to suppress signal from the outer volume 14. The slice selection gradient for the 90° excitation defines a plane that is tilted with respect to the 180° refocusing plane. The volume that experiences excitation and refocusing is thus restricted in one direction – chosen to be the PE direction. The edges of this volume taper in the PE direction and, to provide a rectangular profile, saturation slabs are applied perpendicularly to the 180° plane. This 180° plane defines the orientation of the imaging plane. The angle of the tilted 90° plane (determined by the slice thickness and the PE FOV) was approximately 8° for TFOV D and 14° for TFOV S with respect to the 180° plane. In TFOV D, the PE direction was chosen to be in the SI direction and, in TFOV S, the PE direction was in the LR direction. Specifically, for a coronal plane imaged with TFOV D, the slice selection gradients were applied in the anterior–posterior (AP) direction for the 180° pulse and in the AP/SI direction (with a small component in the SI direction) for the tilted 90° pulse. For TFOV S, the slice selection gradients were applied in the AP direction for the 180° pulse and in the AP/LR direction (with a small component in the LR direction) for the tilted 90° pulse. The saturation slabs are positioned to define the superior and inferior edges of the imaged FOV for TFOV D, or the left and right edges in TFOV S. Differing planning geometries resulted in different readout durations, which were 81 ms and 106 ms for TFOV D and S, respectively. The matrix size for both TFOV D and S was 112 × 112.

A comparison with a conventional full-FOV DTI scan was demonstrated with additional scans on three (of the 12) subjects, who were each scanned with three sequences: (i) a conventional full FOV; (ii) TFOV D; and (iii) TFOV S. TFOV D and TFOV S parameters were identical to the previous scans in all 12 volunteers, but with only four slices to reduce the total scan time for all three scans. The full-FOV scans were imaged with the following parameters: TR/TE = 5000/80 ms; FOV = 380 × 380 mm^2^; voxel size, 3 × 3 mm^2^; SI PE direction (with saturation pulses in the SI direction); slice thickness, 5.5 mm; three signal averages; angulated coronal plane; sensitivity encoding (SENSE) (with acceleration factor of 1.6 in the SI direction). A 16-channel (eight anterior, eight posterior) coil configuration was used. A subject with a kidney cyst was included to demonstrate the ability of TFOV to depict renal cysts.

All scans were performed during free breathing. To minimize motion artefacts, a navigator-triggered sequence was used in which a pencil beam navigator was positioned over the lung–liver interface, with a 5-mm triggering window in the expiration phase of the respiratory cycle. The navigator used a spiral radiofrequency (RF) excitation to excite a ‘pencil beam’ through the diaphragm with real-time profile reconstruction and signal correlation to determine the time of triggering. (There was no technical development made on the navigator itself, although adjustments were made to the timings of the diffusion sequence in order to acquire all four slices immediately after each trigger). The slices were imaged in interleaved sets to prevent slice cross-talk. Each sequence from the first set of scans with 12 subjects (scanned with TFOV D and S) had a nominal scan time of 8.3 min (approximately 15 min including respiratory gating efficiency). Each sequence from the second set of scans with three subjects and a reduced number of four slices (scanned with full FOV, TFOV D and TFOV S) had a nominal scan time of 4.15 min. The time interval between the first and second sets of scans was 5–6 months. Partial Fourier was avoided in this study to minimize the effect of motion during diffusion sensitization, which could cause the *k*-space peak to move outside of the sampled region of *k* space.

An anatomical scan was performed in each subject, acquired with a balanced gradient echo preceded by an inversion pulse. An inversion time of 1320 ms was used to maximize contrast between the kidney medulla and the cortex. All anatomical scans were acquired in a single 12-s breath hold and imaged with the following parameters: TR/TE = 2.4/1.2 ms; FOV = 40 × 40 cm^2^; slice thickness, 5.5 mm; eight contiguous slices; voxel size, 1.8 × 1.8 mm^2^.

### Image reconstruction and analysis

Prior to averaging of the individual diffusion-weighted images, affine registration was performed on the images using the FMRIB Software Library (FSL) (http://fsl.fmrib.ox.ac.uk/fsl/fslwiki/FLIRT) 16, with correlation ratio cost function and trilinear interpolation, to correct for residual motion and eddy current-induced misregistration effects. As the spinal cord is relatively stationary and may be a confounding factor if registered with the kidneys, the images were first cropped manually and the kidneys were separately registered to exclude the spinal cord from registration. Although the effects of in-plane motion were minimized by avoiding partial Fourier sampling and by performing image registration on magnitude images, any through-slice motion results in signal loss. Thus, images were excluded from the analysis based on the peak signal in *k* space. First, the peak *k*-space value was computed for each diffusion-encoding direction and each signal average. Images with peak *k*-space intensities below one standard deviation of the mean were excluded from the analysis. This study used an oversampled number of diffusion directions with multiple averages, which allowed the computation of FA even though some images were excluded. Parts of the image reconstruction process were performed using the MRecon framework (GyroTools LLC, Zurich, Switzerland), which is a software for the reconstruction of images from raw *k*-space data. In this study, it was used to parse the raw data and to grid *k*-space data (which included non-uniform ramp sampling). The MRecon tool was also used to perform SENSE reconstruction (in the full-FOV comparison scans) using coil sensitivity information obtained from a separate reference scan.

The apparent diffusion coefficient (ADC), FA, colour FA (cFA) and diffusion tensors were computed for TFOV D and S. To provide qualitative differentiation between the medulla and cortex, an automatic thresholding algorithm was used to generate maps of FA > 0.3 and FA < 0.25, with the threshold values based on literature values for the medulla (0.21 ± 0.02) and cortex (0.36 ± 0.03) 4. For quantitative assessment, regions of interest (ROIs) were manually drawn over the medulla and cortical regions in each slice (based on the FA map, ADC map and the anatomical scan, and reviewed by a radiologist). To assess the effect of increasing the number of signal averages on the variability of FA, subsets of the full number of averages were reconstructed. For each subject and each subset of averages, the variance of FA within the medulla and the cortical ROIs was computed. Finally, the mean FAs in the medulla and cortex were computed over all imaged slices for both TFOV D and S. Because of non-Gaussian distributions, the Wilcoxon rank sum test was used to compute the statistical significance of FA values between the medulla and cortex, and between TFOV D and S. The medulla and cortical FA values were also computed for the three subjects who were scanned with three different sequences (full FOV, TFOV D and TFOV S).

A signal-to-noise ratio (SNR) analysis was performed on the three subjects who were scanned with all three FOVs. As there was no background region from which the standard deviation of noise could be derived from the TFOV scans, pure noise images were additionally obtained to compute the SNR 17,18. In each of the three subjects additionally scanned with three different FOVs, a pure noise image was acquired by turning off all RF pulses. The SNR was computed based on the NEMA standard MS 1–2001 17 for each type of scan (full-FOV, TFOV D and TFOVS). The SNR was calculated as the mean signal from all diffusion-encoding directions (excluding the *b* = 0 s/mm^2^ image) divided by the standard deviation of the corresponding noise in the same ROI over the kidneys, assuming approximate Rayleigh distribution of noise, as in Dietrich *et al*. 18.

In addition, images were reconstructed before and after image registration. FA maps were computed for each. Intensity was plotted to show the degree of alignment among the *b* = 0 s/mm^2^ and diffusion-weighted images before and after image registration.

## RESULTS

**Figure 1:**
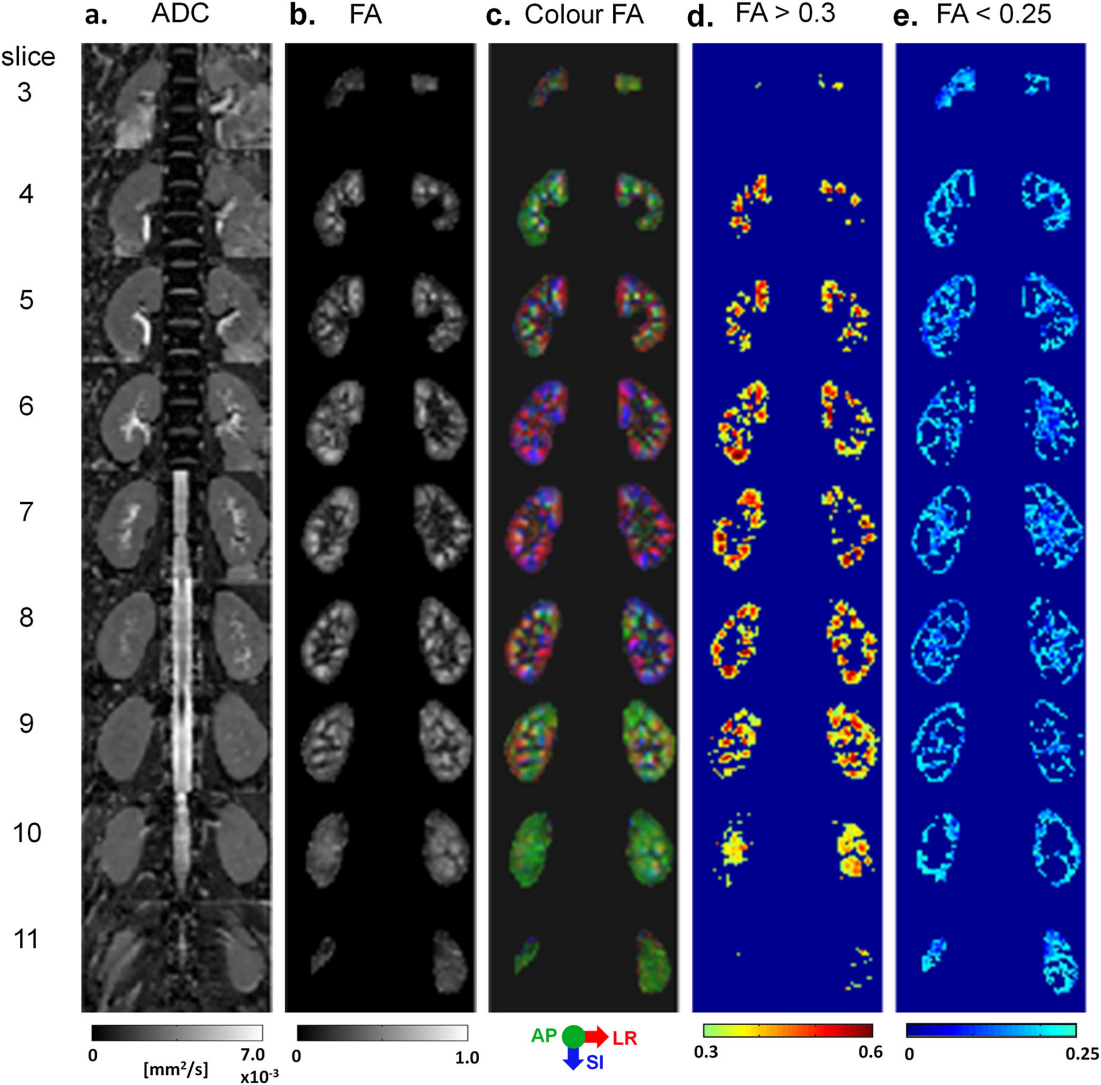
Dual-kidney targeted field of view (TFOV D). Multiple slices throughout the kidneys are shown for: (a) the apparent diffusion coefficient (ADC) map; (b) greyscale fractional anisotropy (FA) map for all kidney voxels; (c) colour FA map for all kidney voxels [red, left–right (LR); green, anterior–posterior (AP), blue, superior–inferior (SI)]; (d) FA map with values > 0.3; (e) FA map with values < 0.25. From subject 3 with a TFOV that measured 220 × 126 mm^2^. Slices 1, 2 and 12 did not include the kidneys and were omitted.

**Figure 2:**
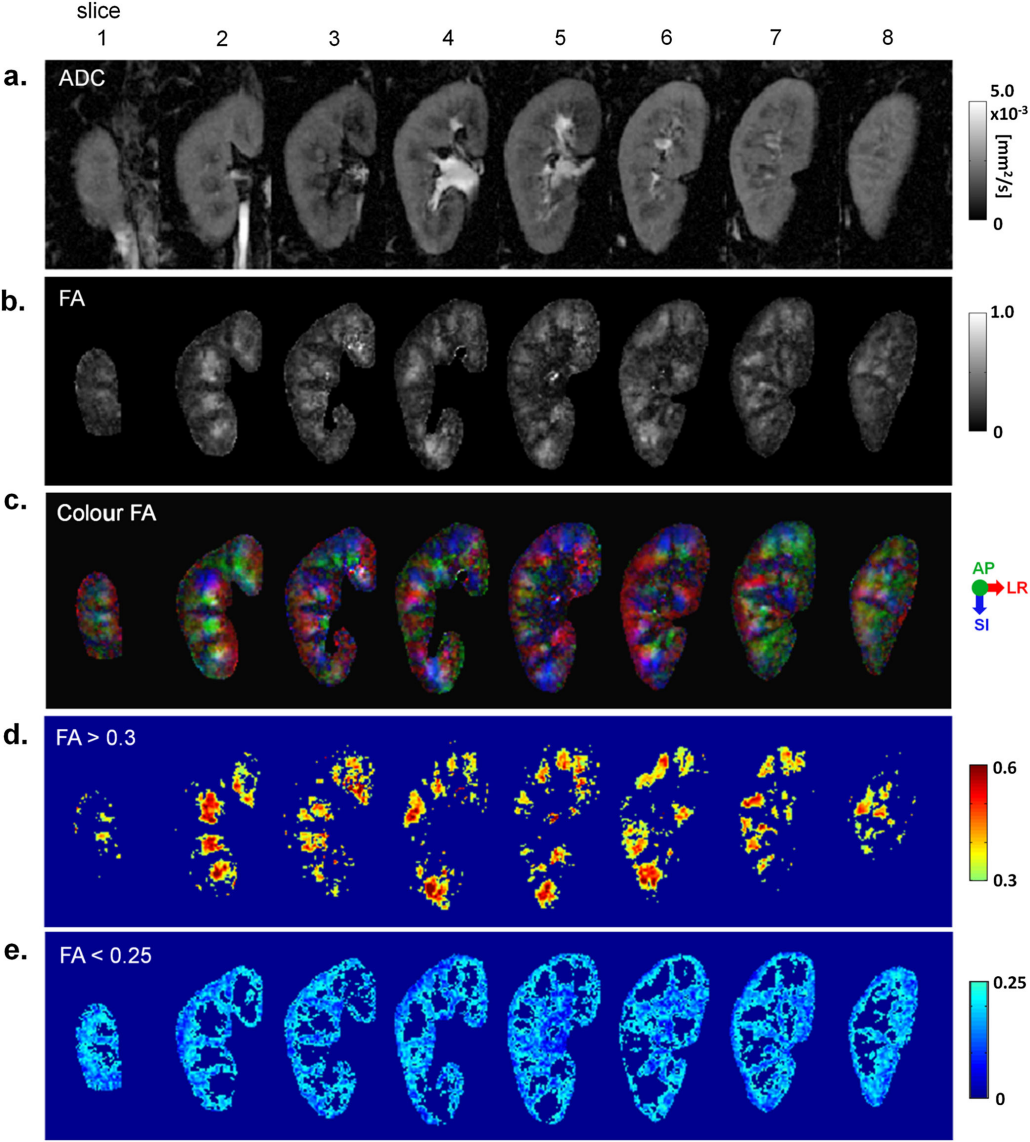
Single-kidney targeted field of view (TFOV S). Eight slices throughout the kidneys are shown for: (a) the apparent diffusion coefficient (ADC) map; (b) greyscale fractional anisotropy (FA) map for all kidney voxels; (c) colour FA map for all kidney voxels [red, left–right (LR); green, anterior–posterior (AP), blue, superior–inferior (SI)]; (d) FA map with values > 0.3; (e) FA map with values < 0.25. From subject 5 with a FOV of 72 × 120 mm^2^.

Figure 1 displays images acquired with TFOV D, including maps of the ADC, FA, cFA, FA > 0.3 and FA < 0.25 of both kidneys. The more anterior and posterior slices are dominated by diffusion orientations in the AP direction, whereas the inner slices consist of more diffusion orientations in the LR and SI directions. The thresholded maps illustrate the locations of the medulla (as seen in the map of FA > 0.3) and cortical regions (included in the map of FA < 0.25). Figure 2 shows the corresponding maps for TFOV S, for one subject in whom the right kidney was imaged.

**Figure 3:**
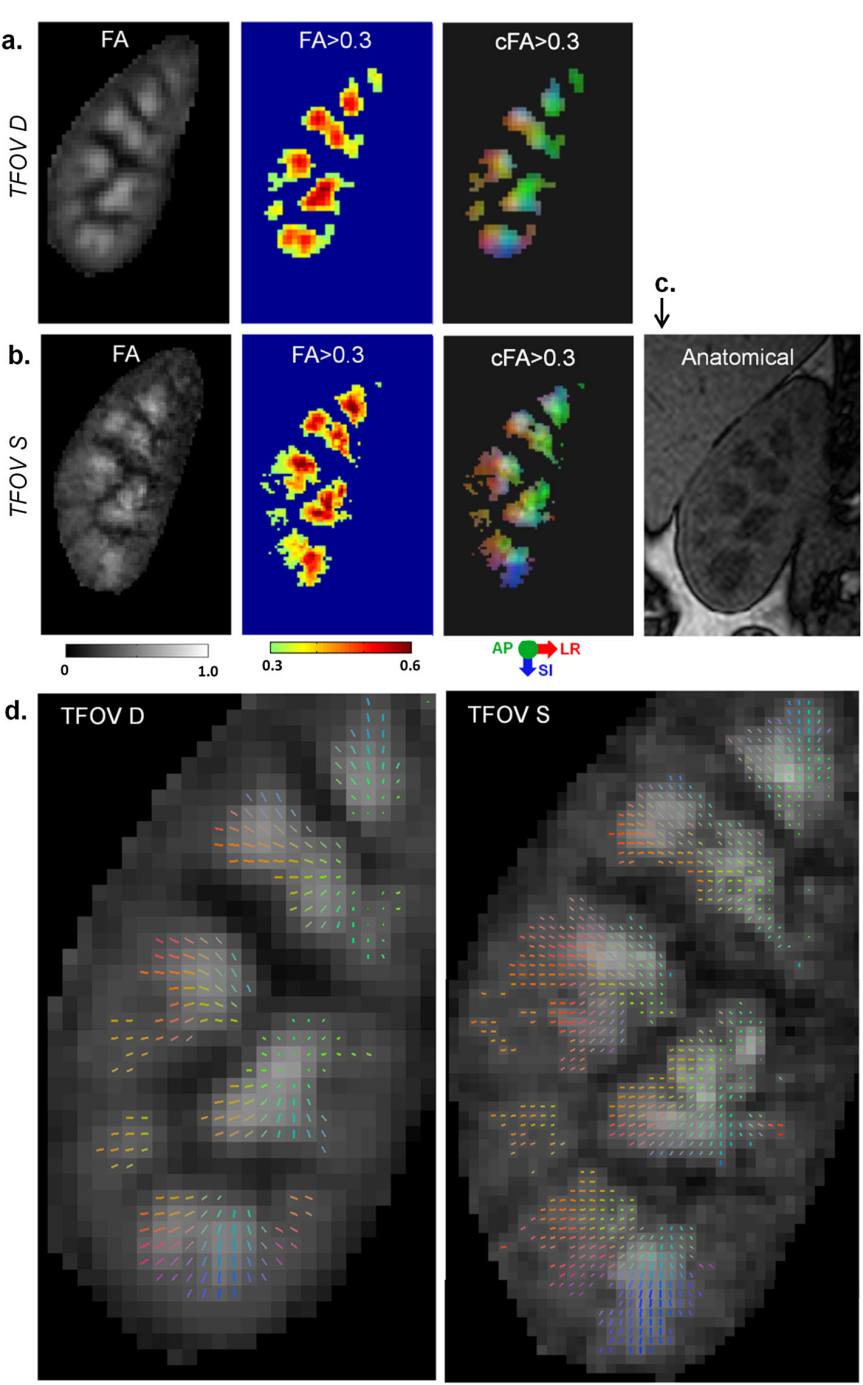
Comparison of dual- and single-kidney targeted field of view (TFOV D and S). A single slice is shown from subject 2. (a) Fractional anisotropy (FA) maps including greyscale FA, FA > 0.3 and colour FA > 0.3 of the right kidney imaged with TFOV D. Only the right kidney that has been imaged by both TFOV D and S is shown. (b) Corresponding FA maps from TFOV S. (c) Closest slice on the anatomical scan showing corticomedullary differentiation. (d) Two-dimensional diffusion tensors are shown for TFOV D and S for the same slice. The colour of the tensors indicates the eigenvector direction in three dimensions with respect to the patient coordinate system [red, left–right (LR); green, anterior–posterior (AP), blue, superior–inferior (SI)]. Tensors have been scaled by the FA and overlaid onto the greyscale FA image.

Consistency between TFOV D and S in the cFA and diffusion tensors can be seen in a representative slice in Fig. 3. The kidney that was imaged by both TFOV D and S was compared in terms of the greyscale FA maps, thresholded FA and thresholded cFA in Fig. 3a, b. The medulla regions correspond to the darker regions seen in the anatomical gradient-echo scan in Fig. 3c. A comparison of the diffusion tensor maps is shown in Fig. 3d. All images show good correspondence between TFOV D and S, including similar medulla regions (as delineated by thresholding) and spatial variation of the diffusion directions (as indicated by the cFA maps and by the vector directions in the diffusion tensor maps). The radial diffusion pattern is evident in the tensor maps, and is seen at higher resolution in TFOV S relative to TFOV D.

**Figure 4:**
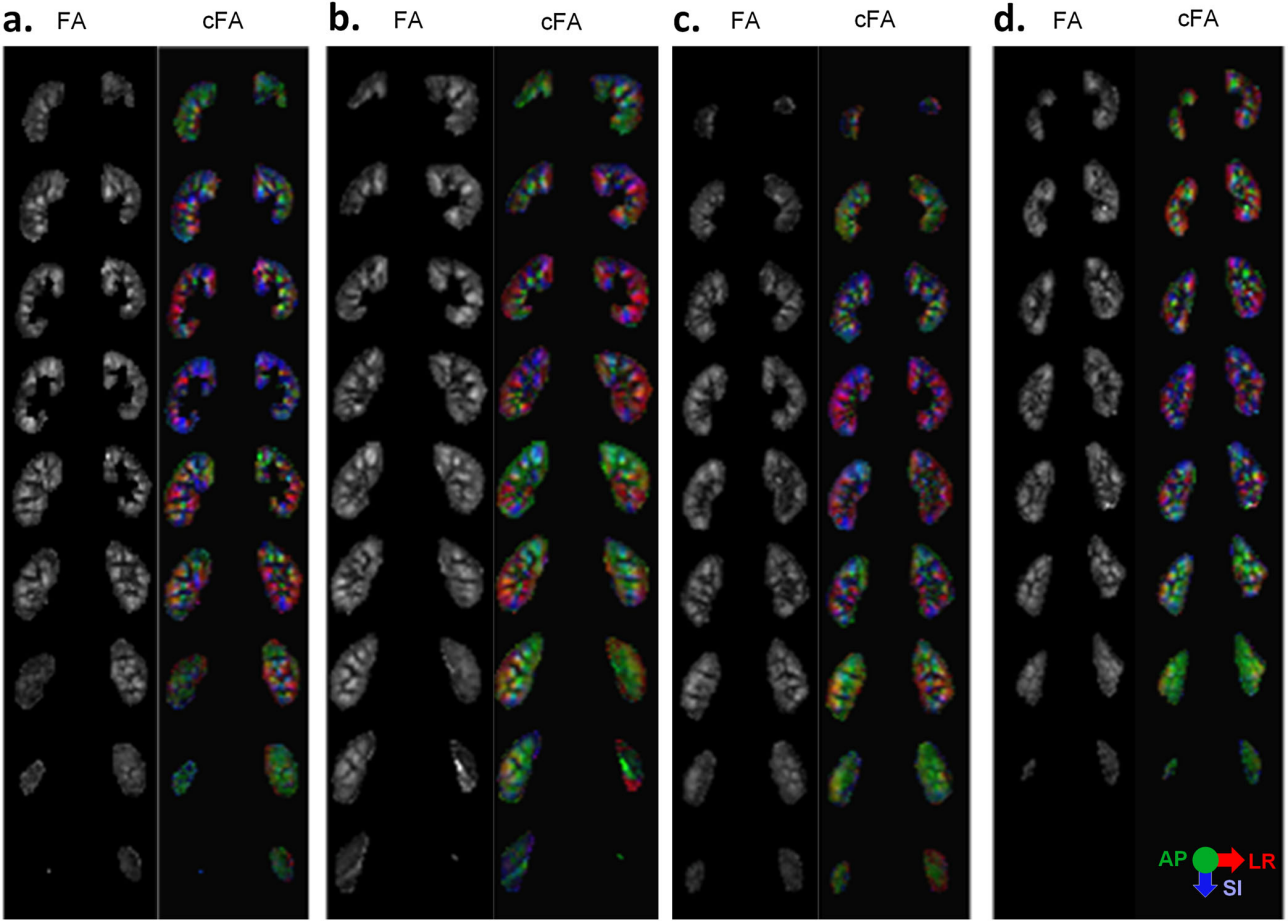
Dual-kidney targeted field of view (TFOV D) for multiple subjects. (a–d) Slices 3–11 of the kidneys are shown for four subjects 1,2,6,7. For each subject, the greyscale fractional anisotropy (FA) and colour FA (cFA) images are shown. Both the medulla and cortical regions are included. The cFA map is scaled by the FA values.

**Figure 5:**
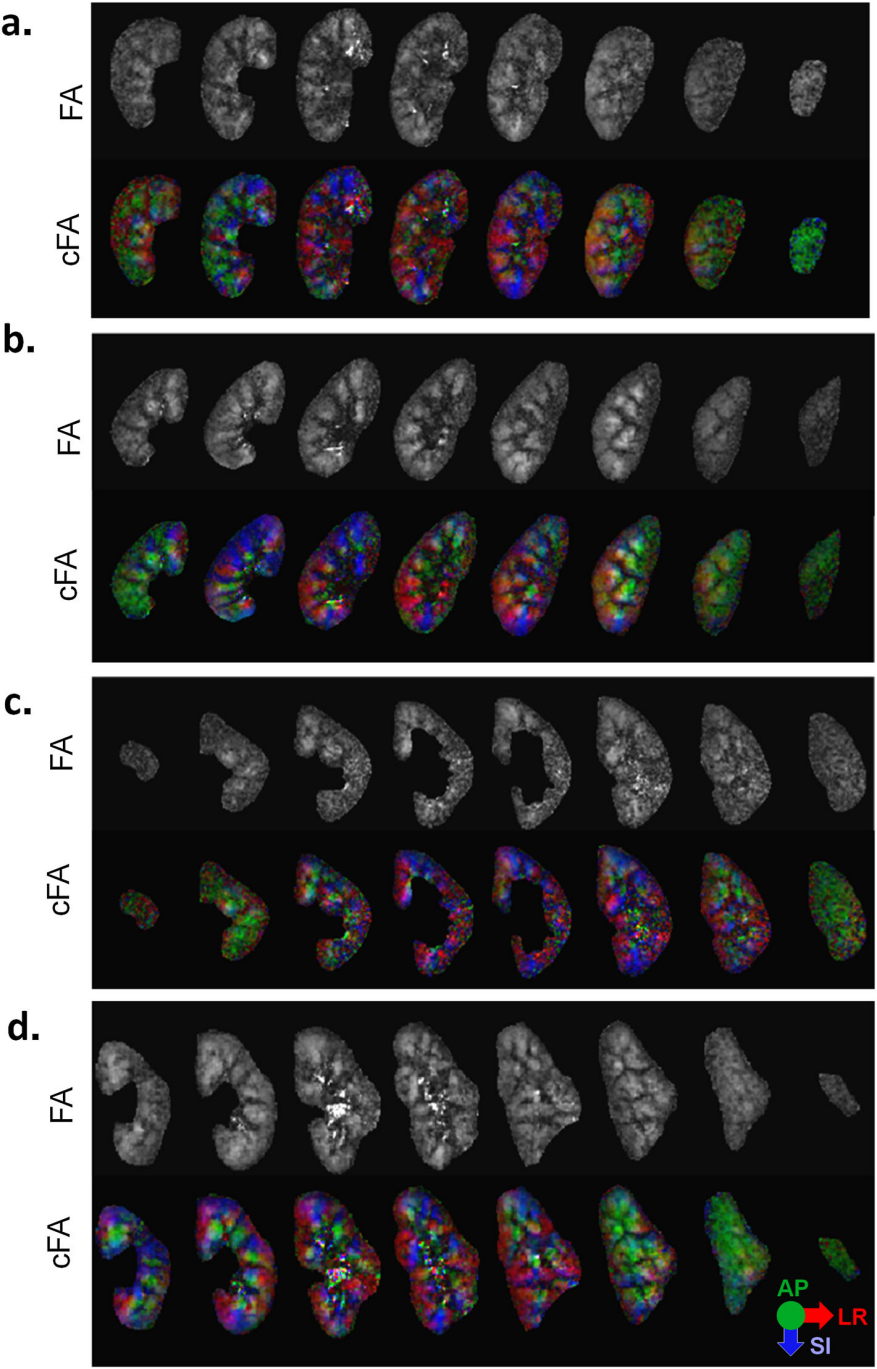
Single-kidney targeted field of view (TFOV S) for multiple subjects. (a–d) A set of eight slices of the kidneys are shown for four subjects 1,2,6,7. The right kidney was scanned in subjects 1 and 2, whereas the left kidney was scanned in subjects 6 and 7. The greyscale fractional anisotropy (FA) and colour FA (cFA) images are shown.

**Figure 6:**
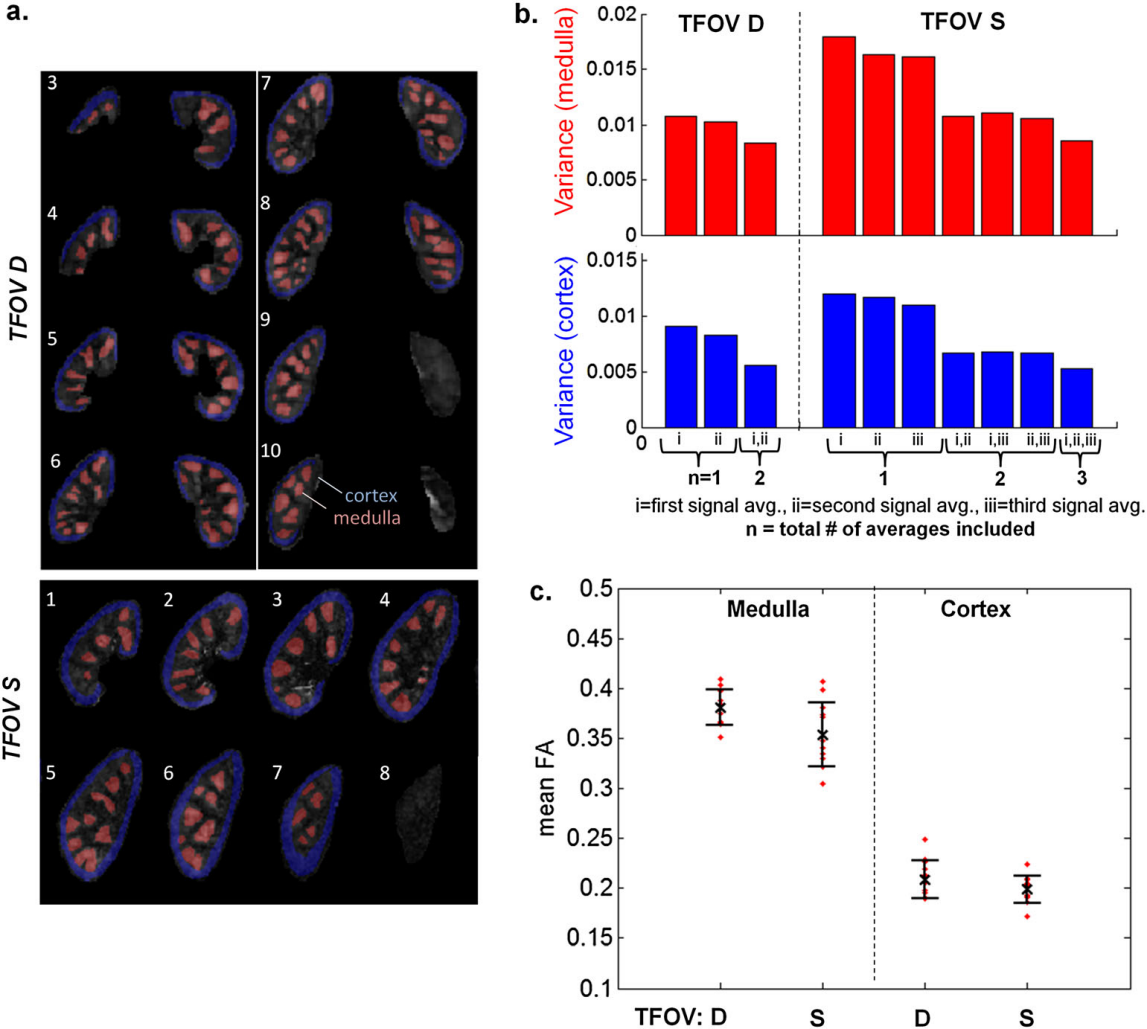
Global assessment of fractional anisotropy (FA) in the medulla and cortex. (a) Regions of interest (ROIs) of the medulla (red) and cortex (blue) for one subject for dual-kidney targeted field of view (TFOV D) and single-kidney targeted field of view (TFOV S). (b) The mean variance of the medullary and cortical FA values for all possible subsets of the total number of signal averages used in image reconstruction, showing the effect of increasing averages. The height of each bar represents the mean variance of each subset, where the variance was computed for each subject over medulla/cortical ROIs and then averaged over all subjects. Each subset consists of combinations of the first average labelled ‘i’, the second average ‘ii’ and the third average ‘iii’. (c) The mean medullary and cortical FA values over 12 healthy subjects, globally quantified over all slices of the kidneys, for both TFOV D and S (using all signal averages in the image reconstruction).

FA and cFA maps are shown for several volunteers in Figs. 4 and 5. Both maps show similar patterns throughout the kidney volume, with diffusion orientations predominantly in the AP direction in the peripheral slices, and in the LR and SI directions in the central slices. Figure 6a displays manually drawn ROIs for the medulla and cortex for one subject, which were used as quantitative measures of FA. The quantitative results in Fig. 6b show the effect of increasing signal averages. In all cases, there is a decreasing trend in the mean variance as signal averages are added. In Fig. 6c, the FA values in the medulla and cortex are plotted for both TFOV D and S, and averaged over all subjects. The mean medulla and cortical FA values were 0.38 ± 0.017 and 0.21 ± 0.019, respectively, for TFOV D, and 0.35 ± 0.032 and 0.20 ± 0.014, respectively, for TFOV S, using all averages. For the same number of (two) signal averages, there was no significant difference between TFOV D and S for the medulla or cortical FA (*p* > 0.05 for both, with *p* = 0.14 for the medulla and *p* = 0.10 for the cortex). Including the third signal average in TFOV S slightly lowered the medulla FA with a small but significant difference (*p* = 0.030). There was a significant difference in FA between the medulla and cortex (*p* < 0.001 for both TFOV D and S).

**Figure 7:**
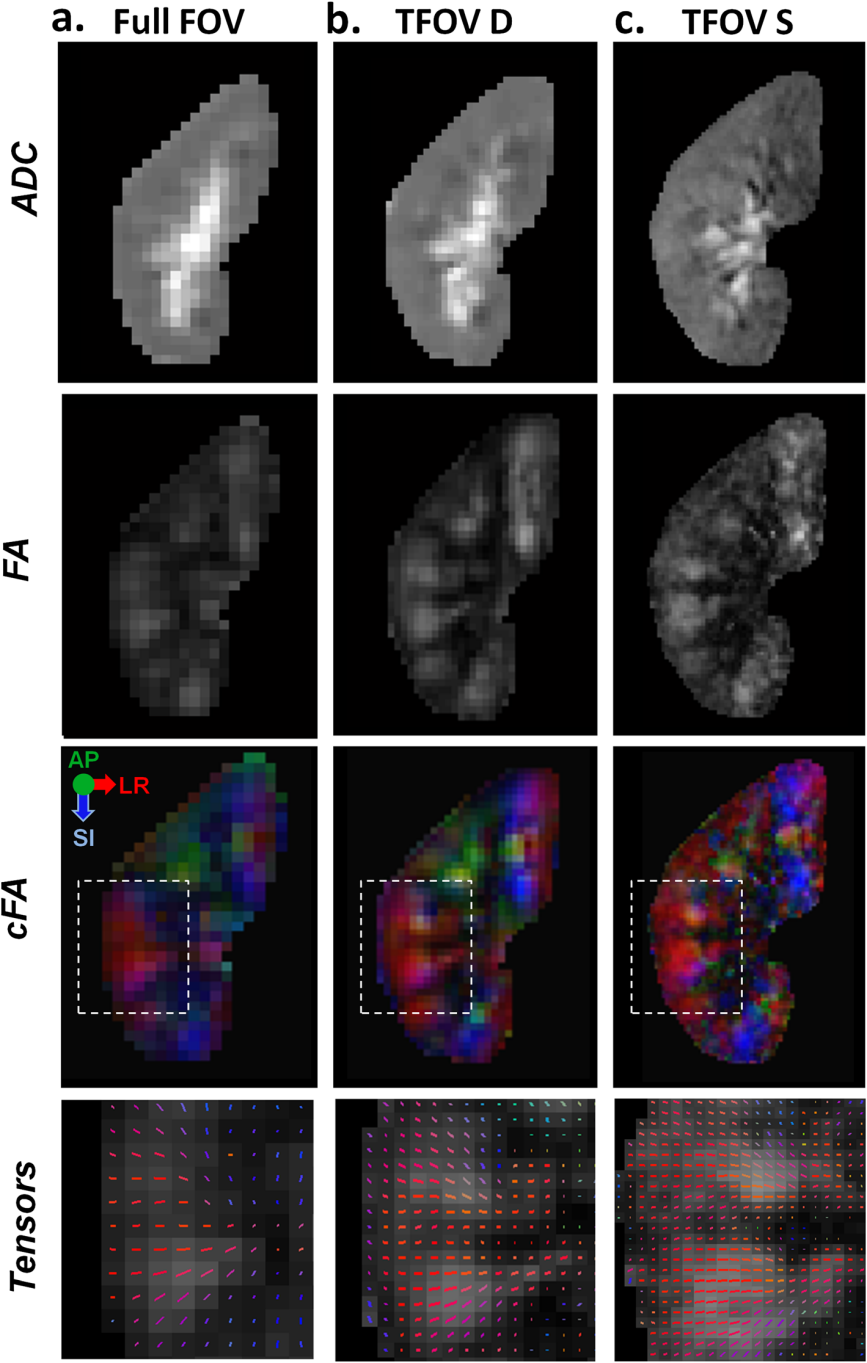
Comparison with full field of view (FOV). An example slice is shown in which a subject was imaged with three different FOVs: (a) full FOV with 3 × 3-mm^2^ in-plane voxel size; (b) dual-kidney targeted FOV (TFOV D) with 2 × 2-mm^2^ in-plane voxel size; (c) single-kidney targeted FOV (TFOV S) with 1.2 × 1.2-mm^2^ in-plane voxel size. The apparent diffusion coefficient (ADC), fractional anisotropy (FA), colour FA (cFA) and a zoomed-in view of the diffusion tensor orientations are displayed. The tensors are displayed for the regions of interest (ROIs) shown on the cFA images.

**Figure 8:**
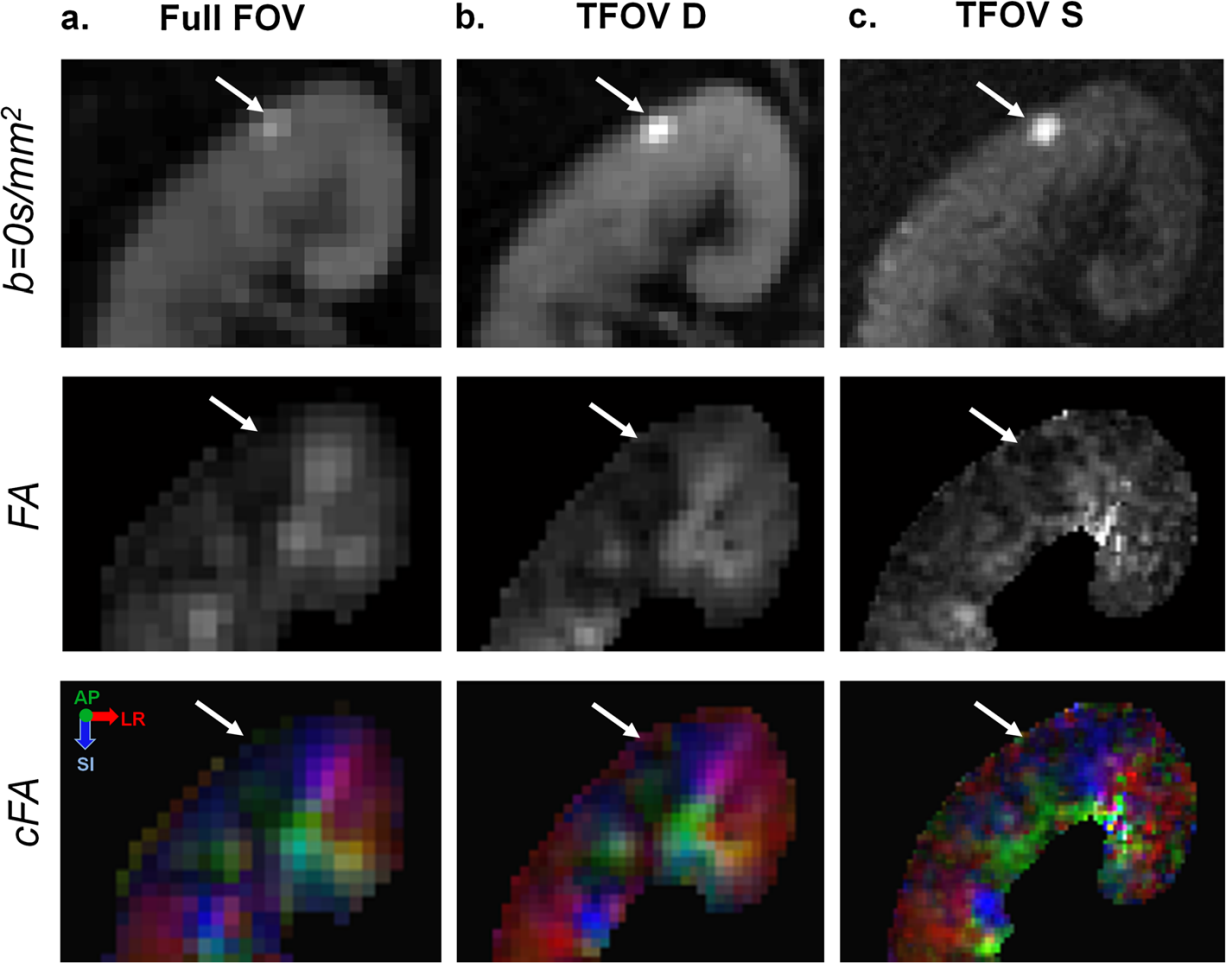
Example of renal cyst. An example of a small renal cyst is shown for all three fields of view (FOV): (a) full FOV; (b) dual-kidney targeted FOV (TFOV D); (c) single-kidney targeted FOV (TFOV S). The *b* = 0 s/mm^2^ images, fractional anisotropy (FA) maps and colour FA (cFA) maps are shown. White arrows in each image point to the small cyst.

Higher resolution with TFOV D and S offered improved depiction of structures within the kidney on the ADC map compared with the full-FOV scan (as shown in Fig. 7). The resulting diffusion tensors were consistent (as shown in the tensor map in Fig. 7, as well as in the cFA maps in Figs. 7 and 8). In the subject with a small renal cyst, high-resolution FA maps of the cyst were reliably obtained. Qualitative comparison showed better depiction of the cyst boundaries in higher resolution TFOV diffusion scans relative to lower resolution full-FOV scans (as shown in Fig. 8). Clear delineation of the boundaries of the cyst can be seen in the FA map.

**Figure 9:**
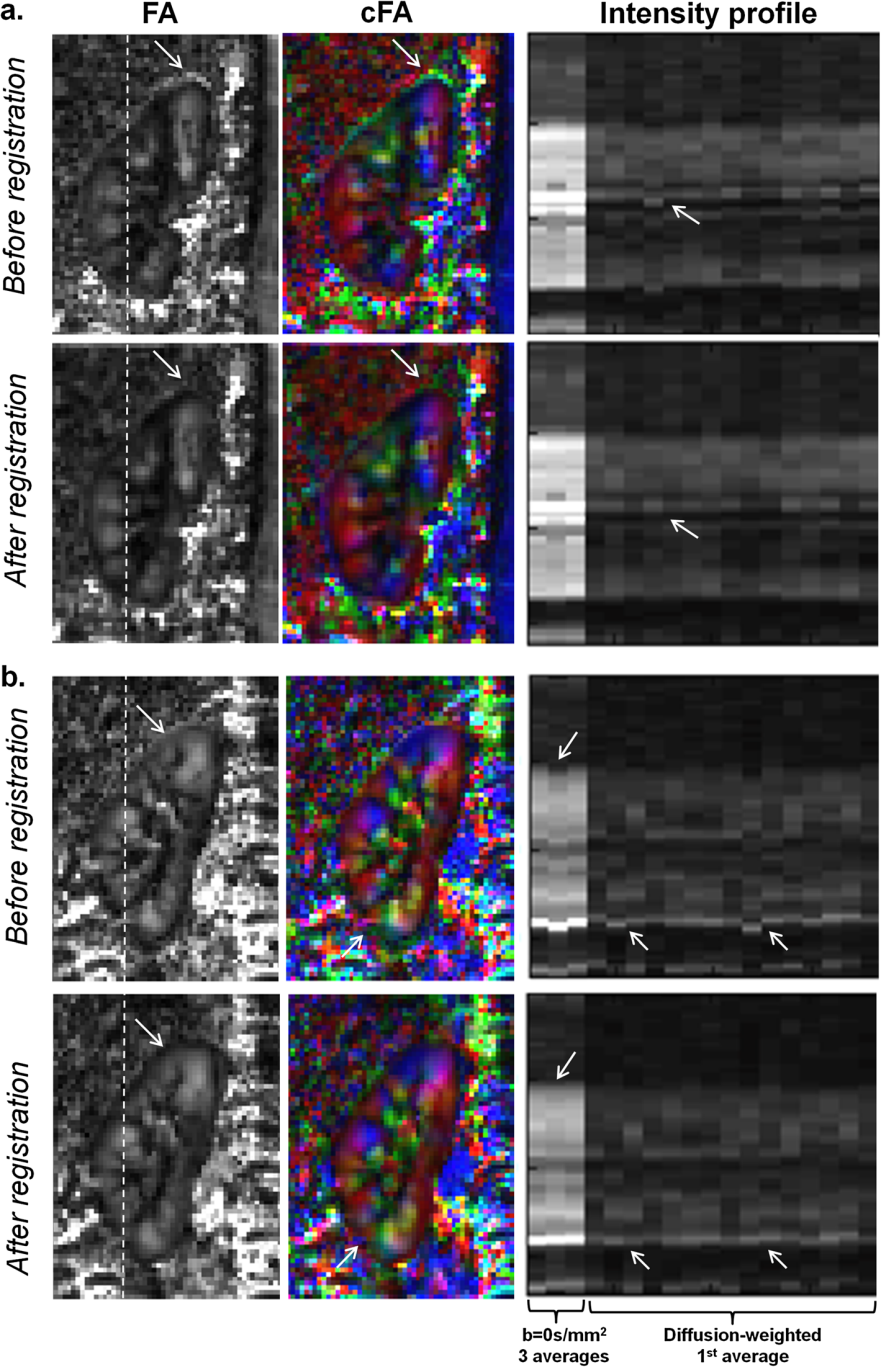
Before and after image registration. Fractional anisotropy (FA) and colour FA maps are shown before and after affine image registration. Maps are shown for: (a) a subject in whom there was relatively little respiratory motion; (b) a subject in whom there was more substantial motion. Images shown here were acquired with dual-kidney targeted field of view (TFOV D). Both scans had navigator triggering with a pencil beam navigator. Intensity line plots are shown for the *b* = 0 s/mm^2^ and diffusion-weighted images. The locations of the intensity plots are shown by the dotted white lines on the FA images. Arrows indicate areas in which image registration resulted in reduced misalignment artefacts.

Figure 9 displays images in two subjects to compare FA maps before and after image registration. The first subject (Fig. 9a) had relatively little motion from respiration, whereas the second (Fig. 9b) had comparatively more motion. Regions of the image that benefit from affine image registration are indicated by the arrows in Fig. 9. The differences in the FA and cFA maps are mainly seen at the edges of the kidney.

**Figure 10:**
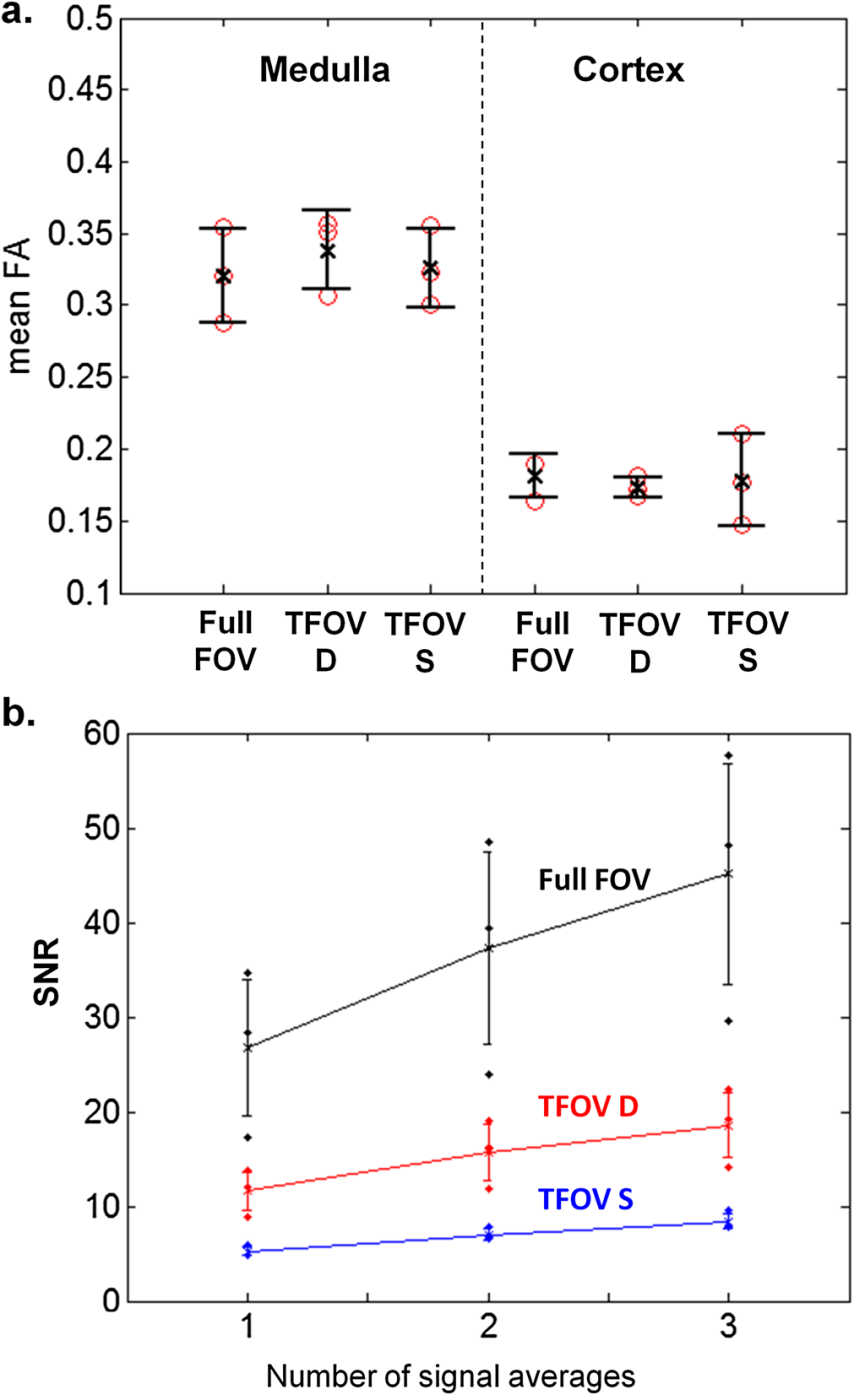
Fractional anisotropy (FA) values and signal-to-noise ratio (SNR) analysis for comparison scans. (a) Medulla and cortical FA values for three subjects imaged with three sequences [full field of view (full FOV), dual-kidney targeted FOV (TFOV D) and single-kidney targeted FOV (TFOV S)]. FA values were derived from images reconstructed from all three signal averages. (b) SNR plots for the same three subjects. Means and standard deviations (over the subjects) are shown, as well as the individual SNRs for each scan of each subject. Images were reconstructed with different numbers of signal averages 1–3 for the SNR comparison.

The plots in Fig. 10a show the medulla and cortical FA computed from the three subjects who were scanned with three sequences. For the full-FOV, TFOV D and TFOV S scans, the mean medulla FA values were 0.32 ± 0.027, 0.34 ± 0.022 and 0.33 ± 0.023, and the mean cortical FA values were 0.18 ± 0.012, 0.17 ± 0.006 and 0.18 ± 0.026, respectively. Results from the SNR analysis in Fig. 10b showed that the full FOV (with the lowest spatial resolution) had the highest SNR, followed by TFOV D, with the lowest SNR for TFOV S (with the highest spatial resolution). Using all three averages, the SNR was 45.2 ± 11.7 for full FOV, 18.6 ± 3.4 for TFOV D and 8.4 ± 0.8 for TFOV S. An explanation for the wider standard deviation is that there were fewer voxels in the kidney regions per subject as the voxel size increased. The SNR in the full-FOV scan is about five to six times higher than that of the smallest FOV.

## DISCUSSION

The results show high consistency in the FA maps and colour-coded diffusion orientations across subjects. Consistent results in FA between TFOV D and S were demonstrated both qualitatively and quantitatively. There was agreement between TFOV D and S despite different PE directions and image resolutions. Residual differences could be caused by differing numbers of signal averages and bandwidths. Although TFOV D and S showed comparable mean FA values, the increased resolution in TFOV S (relative to TFOV D) offered an improvement in the visualization of diffusion tensors. In certain situations, it could be advantageous to image one kidney with higher resolution, for example, in patients with a resected kidney or in a single-kidney follow-up protocol. The ability to image contiguous slices throughout the kidney allowed the global assessment of FA throughout the entire kidney.

Lower FA values were seen with three averages relative to two averages in TFOV S. If the true underlying FA is low, noise would result in higher FA. The addition of a signal average increases the SNR. This is speculated to be the reason why FA is lower and more accurate with three signal averages. This is consistent with the trend seen on going from one to two signal averages, where FA was significantly lowered after the addition of the second average. However, other factors may have led to different FA values, including the possibility of motion occurring during the diffusion pulses. The results in this study suggest that a minimum of two averages should be used for DTI. However, this choice is dependent on the set of coils, patient geometry and overall SNR available for each acquisition.

It is possible to minimize motion by breath-hold acquisitions. However, the image resolution and quality achieved within a single breath hold 19 would be limited, and multiple breath holds would require considerable patient co-operation. Here, a free-breathing, navigator-triggered acquisition was chosen. Results from the evaluation of subsets of averages suggest that the total scan time could be shortened for a small compromise in image quality.

An alternative method of multi-slice TFOV has been demonstrated for spinal imaging in Jeong *et al*. 12. The scheme in Jeong *et al*. 12 relies on the use of an extra refocusing pulse at the end of the echo planar image readout to re-invert spins in the out-of-slice regions. This may be sensitive to through-slice motion between refocusing pulses and imperfections in these pulses. In our application, in which respiratory motion can be a problem, we chose a TFOV approach using non-coplanar application of excitation and refocusing pulses, combined with outer volume suppression 13,14.

The study used only one *b* value, which is in part because of the limited scan time per session and the need to perform multiple TFOV scans. For the purposes of obtaining diffusion tensor orientations, one intermediate *b* value is sufficient. Higher *b* values can allow for greater sensitivity to the tensor directions (for example, for fibre tracking), but also result in a lower SNR. Thus, an intermediate *b* value of 450 s/mm^2^ was chosen to balance these trade-offs. The chosen *b* value is also above the perfusion-sensitive range 20.

For the full-FOV comparison, parallel imaging was applied using a SENSE factor of 1.6. Parallel imaging is less effective in small fields of view because of the lack of coil sensitivity variation over these smaller FOVs. In terms of reductions in overall scan time, much larger reductions could be achieved in the future by using multi-band slice excitation techniques, which take advantage of coil sensitivity variation in the through-slice direction.

Differences in the FA maps were seen mostly at the edges of the kidneys before and after image registration, with little change in the central parts of the kidney. Although image registration resulted in improvement, relatively minor differences before and after registration suggest that the pencil beam diaphragm navigator with a 5-mm gating window was robust in eliminating most effects of motion during free breathing.

The TFOV approach used in this study was successful in suppressing signal from outside of the planned FOV. This may be useful in larger patients, as there is no requirement to change the size of the planned FOV to match the patient size.

Our study had several limitations. Relatively thick slices were used to ensure adequate coverage of the kidneys. Although in-plane tensors could be derived accurately, three-dimensional tractography would probably require higher resolution in the slice direction, especially in the kidneys, in which there could be rapid changes in tensor directions over short distances.

Another limitation of the study is that the comparison of the tensors was qualitative. Future work might include the exploration of quantitative methods of describing tensors in kidney DTI. An ‘inter-voxel diffusion coherence index’ was developed by Wang *et al*. 21. However, it is unclear whether this metric would sufficiently reflect differences between healthy and diseased kidneys, given the natural variability of kidney structures and resulting tensor directions across subjects. In addition, the order of TFOV D and S was not randomized in this study. It was assumed that each subject's respiration pattern was similar throughout the scan session and that there was no substantial bulk motion between different types of scans. It is noted that the quality of the comparison full-FOV image could be further improved. For example, Lanzman *et al*. 10 showed greyscale FA maps with superior corticomedullary differentiation compared with our full-FOV images for similar nominal scan times. Direct comparison is, however, difficult because of the differing subject pathologies and parameters used in the study by Lanzman *et al*. 10 (including the use of multiple non-zero *b* values and also 20 diffusion-encoding directions).

One of the major drawbacks of the use of navigator triggering is the extended scan time. Continuous acquisition of data without respiratory gating and with motion correction is a possible alternative approach that could significantly reduce the scan time. However, this would rely heavily on the ability of motion correction by image registration techniques to remove motion artefacts. Image registration has been shown to be suboptimal for diffusion images that have significant contrast changes 22,23. The success of image registration is also dependent on the SNR. It was found that image registration performed better on diffusion images obtained at lower *b* values than on those obtained at higher *b* values when the SNR was low 24. For these reasons, a combination of respiratory triggering and image registration was used in our study. Triggering helped to reduce through-plane motion, which cannot be compensated by two-dimensional registration techniques.

The SNR analysis (performed on three subjects who were scanned with three fields of view including the full-FOV comparison) illustrates an expected loss of SNR when going to higher resolution in TFOV D and S. The ability of high resolution to visualize structures such as small cysts should be balanced against a loss of SNR. This study demonstrates that kidney DTI is feasible at different resolutions using TFOVs, but the appropriate choice of resolution would depend on the size of the relevant kidney structure and the nature of the pathology. Although relatively small structures make up the kidneys, including the medulla pyramids, it remains to be shown whether renal pathologies would benefit from characterization with high-resolution DTI.

Diffusion MRI can be used for the differentiation of benign cystic lesions from cystic renal cell cancers 25 and for the prediction of the histological subtype of renal cell carcinomas (RCCs) 26. Although renal DTI is less commonly used in clinical practice than DWI, FA derived from DTI may be a good indicator of renal function. In patients who have received renal transplants, the medullary FA correlates with renal function 10. FA may be helpful for the characterization of RCCs, cysts and cystic lesions. Notohamiprodjo *et al*. 19 included subjects with simple renal cysts and malignant lesions, and found that the FA values of simple cysts were significantly lower than those of solid tumours. In the same study, RCCs were shown to have a more inhomogeneous pattern and a wider range of FA, possibly owing to the variety of histological subtypes 19. As such, the spatial distribution (and not only the mean value) of FA may be of importance for the differentiation of renal masses 19, and thus high-resolution imaging may be beneficial. It is possible that high-resolution diffusion imaging would allow the better depiction of various types of cysts (especially small ones) and also offer improved imaging of spatial heterogeneity in RCCs.

As diffusion imaging is sensitive to the underlying microstructural changes, it is potentially useful for the non-invasive evaluation of kidney function. Diseases that alter the transport, filtration and re-absorption of water in the kidneys may be reflected in changes in ADC and FA. Changes in diffusion parameters could be an early indication of functional alterations, which could precede structural changes in the kidneys. Although the precise biological mechanisms responsible for changes in FA still need to be established, early DTI studies of renal allografts 9,10 show promise in the evaluation of renal function. The ability to robustly perform diffusion imaging at high spatial resolution is expected to be a beneficial tool for such evaluation.

## CONCLUSIONS

Using a respiratory-triggered, multi-slice, TFOV technique, FA and colour-coded diffusion tensor maps were demonstrated for two implementations of TFOV in which multiple contiguous slices allowed the global analysis of FA. An in-plane spatial resolution of 1.2 × 1.2 mm^2^ was achieved (in TFOV S), which had not been obtained previously in kidney DTI in a free-breathing setting. Colour-coded diffusion orientations and diffusion tensors were shown to be consistent over a number of subjects. High-spatial-resolution DTI is non-invasive and has the potential to lead to improved characterization in kidney disease.

## Abbreviations

ADC: apparent diffusion coefficient
AP: anterior–posterior
cFA: colour FA
DTI: diffusion tensor imaging
DWI: diffusion-weighted imaging
FA: fractional anisotropy
LR: left–right
PE: phase-encoding
RCC: renal cell carcinoma
RF: radiofrequency
RFOV: reduced field of view
ROI: region of interest
SENSE: sensitivity encoding
SI: superior–inferior
SNR: signal-to-noise ratio
TFOV: targeted field of view.
